# Supplementary material for: Antisense oligonucleotide-mediated correction of CFTR splicing improves chloride secretion in cystic fibrosis patient-derived bronchial epithelial cells
Source: Nucleic Acids Res. 2020 Jun 10;48(13):7454–67. doi: 10.1093/nar/gkaa490 (PMC7367209; doi:10.1093/nar/gkaa490)
Supplement: gkaa490_Supplemental_File [file gkaa490_supplemental_file.pdf]

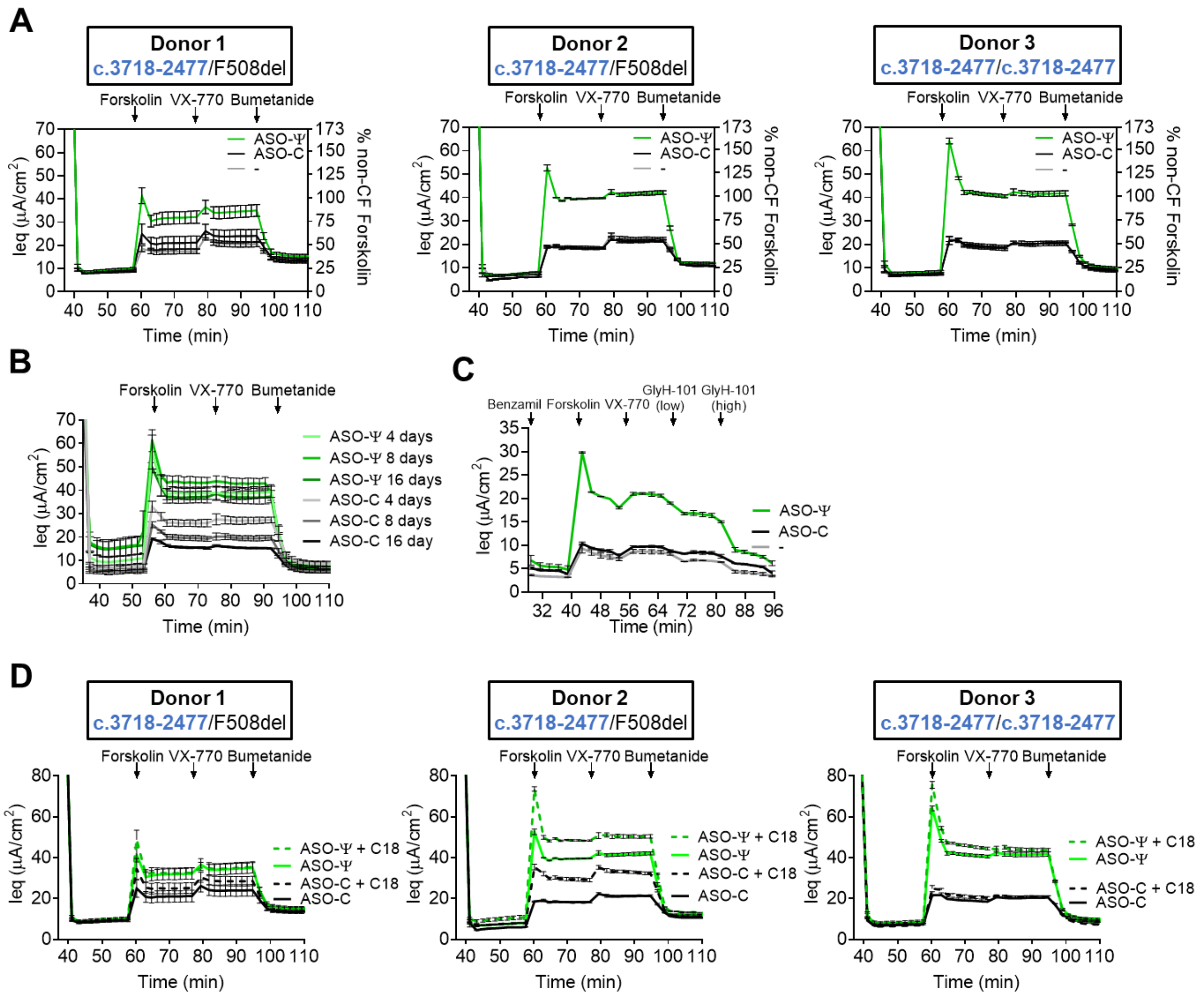

Supplementary Figure S1. Current traces with error bars. (**A**) Current traces from Figure 2 with error bars. (±SEM; N=9 (donor 1), N=3 (donor 2), N=4 (donor 3)). (**B**) Current traces from Figure 4 with error bars. (±SEM; N=3). (**C**) Current trace from Figure 5 with error bars. (±SEM, N=2). (**D**) Current traces from Figure 6 with error bars. (±SEM; N=9 (donor 1), N=3 (donor 2), N=4 (donor 3)).

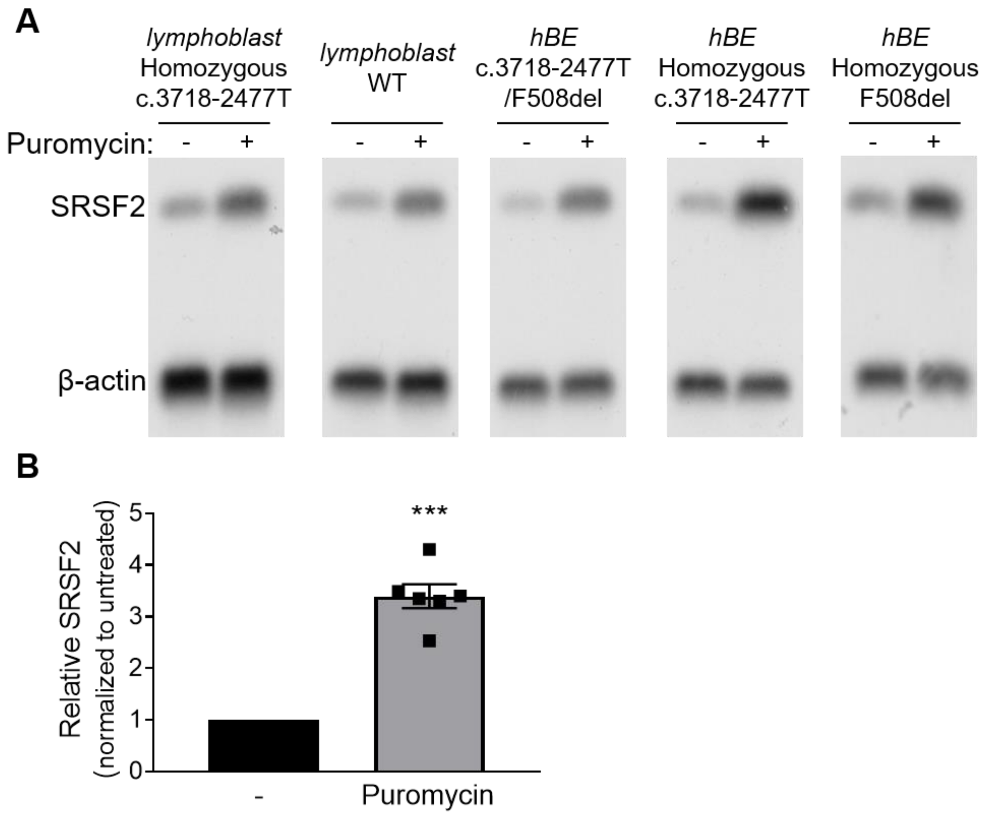

Supplementary Figure S2. Puromycin treatment of lymphoblasts inhibits nonsense-mediated mRNA decay of SRSF2 mRNA isoform with premature termination codon. **(A)** An RT-PCR analysis of SRSF2, a known substrate of NMD, expression in CFTR expressing cell models used in the study treated (+) or untreated (-) with puromycin (200µg/ul) for 9 hours (lymphoblasts) or 6 hours (hBEs). β-actin was used as a control for RNA expression. **(B)** SRSF2 expression after puromycin treatment was quantified and normalized to the untreated samples (±SEM; N=5; one sample t-test, ***p<0.001).

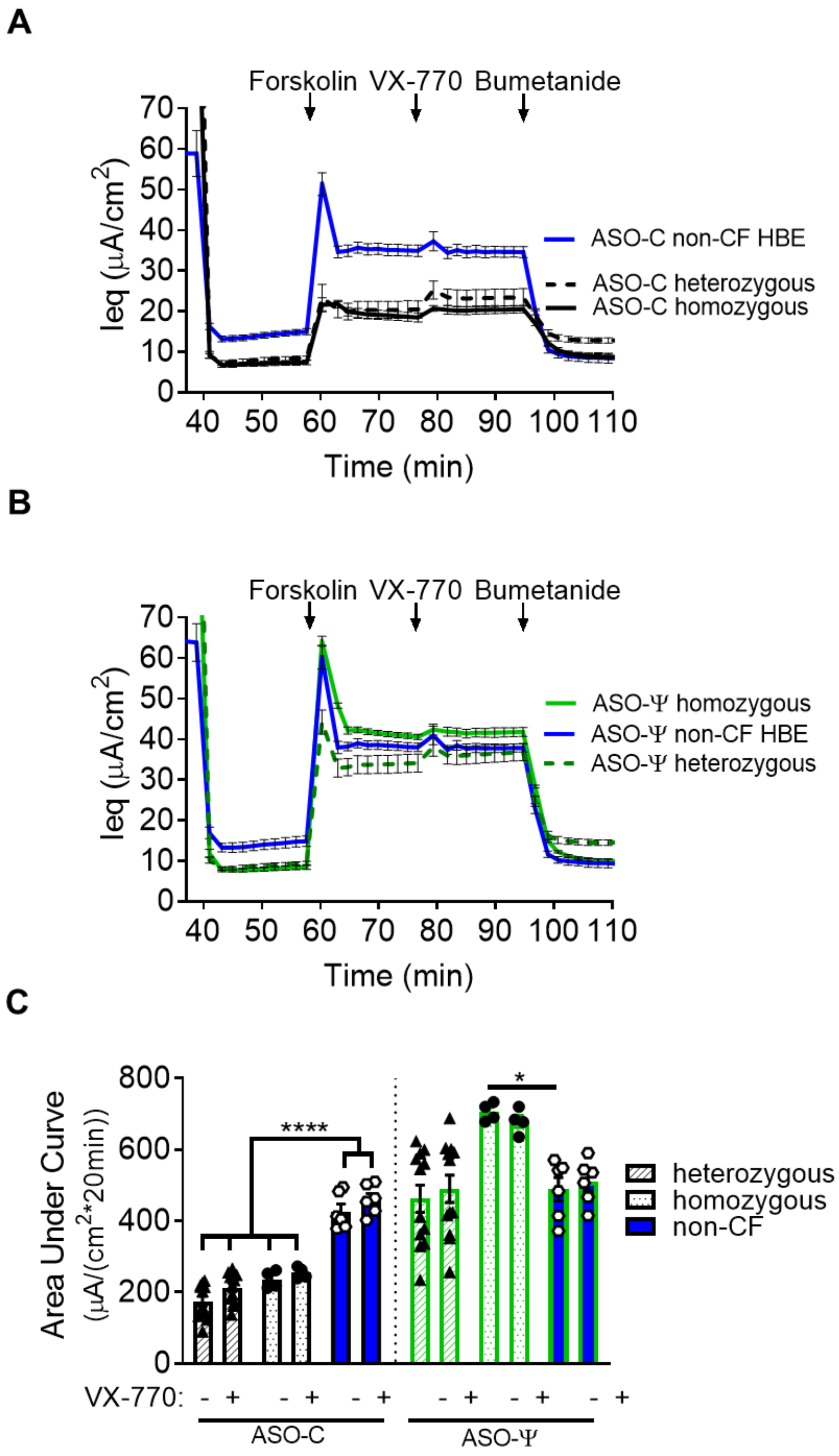

Supplementary Figure S3. Chloride currents in hBE cells from *CFTR* c.3718-2477T donor and non-CF donor cells. **(A)** Average I_{eq} traces from hBE cells from non-CF donors (donor 5 and donor 6) (blue line), compound heterozygous donors 1 and 2 and a

homozygous donor 3 cells treated ASO-C (black lines) and **(B)** ASO-ψ. **(C)** Area under the curve of the forskolin treatment period was quantified for each genotype (±SEM; heterozygous N=12, homozygous N=4, non-CF N=6; One-way ANOVA, Tukey's multiple comparisons test, *p<.05, ****p<0.0001).

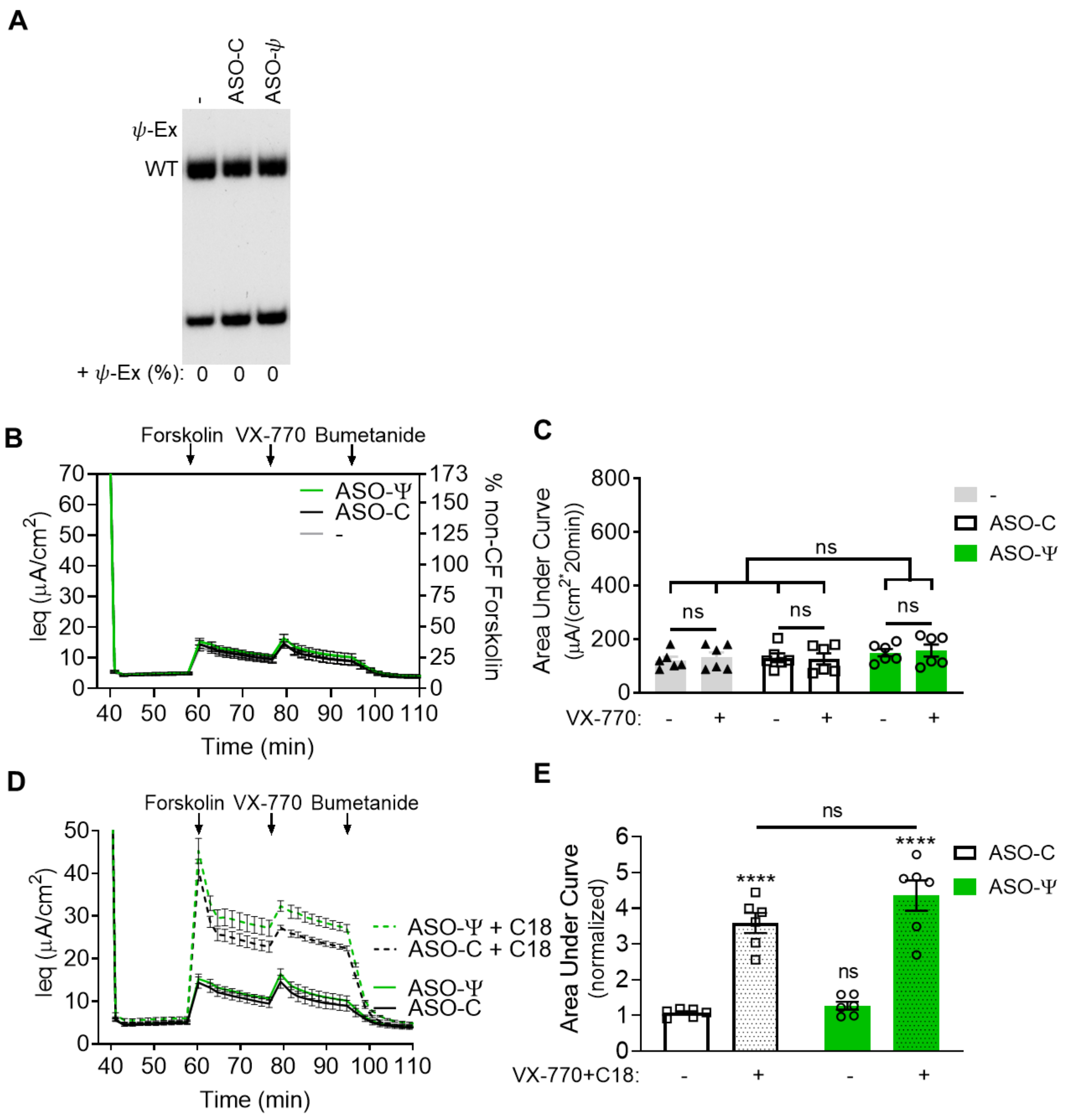

Supplementary Figure S4. Corrector (C18) and potentiator (VX-770), but not ASO-ψ treatment improves chloride channel activity in F508del-*CFTR* cells. **(A)** RT-PCR analysis of homozygous F508del-*CFTR* patient hBE cells (donor 4) treated with vehicle, ASO-C, or ASO-ψ. β-actin was included as a control for total RNA level. Splicing was quantified as the percent of RNA transcripts with the pseudo-exon insert [+ψ-Ex/((+ψ-Ex)+WT)x100)] and is shown below each lane. **(B)** Average I_{eq} traces calculated from the TECC assay (left y-axis) or normalized to percent non-CF average forskolin-specific

currents (right y-axis) (average forskolin-specific I_{eq}=41 μA/cm$^{2;}$ donors 5, 6) from F508del-*CFTR* homozygous hBE cells treated with vehicle (grey lines), ASO-C (black lines), or ASO-ψ (green lines). **(C)** Area under the curve of the forskolin (unhashed) or forskolin + VX-770 (hashed) treatment period was quantified for samples treated with vehicle, ASO-C, or ASO-ψ (±SEM; N=6; One-way ANOVA; Tukey's multiple comparisons test between groups; Sidak's multiple comparisons test within groups, ns=p>0.01). **(D)** Average I_{eq} traces from F508del-*CFTR* homozygous hBE cells treated with ASOs as before and with DMSO (solid lines) or C18 (6 μM, dashed lines). **(E)** Area under the curve analysis of cells treated with (hashed) or without (unhashed) CFTR modulators (C18, VX-770) along with ASO-C (white) or ASO-ψ (green) was quantified. Data was normalized to the quantification for forskolin treatment in vehicle treated samples (±SEM; N=6; One-way ANOVA, Tukey's multiple comparisons test, ****p<0.0001, ns=p>0.01).

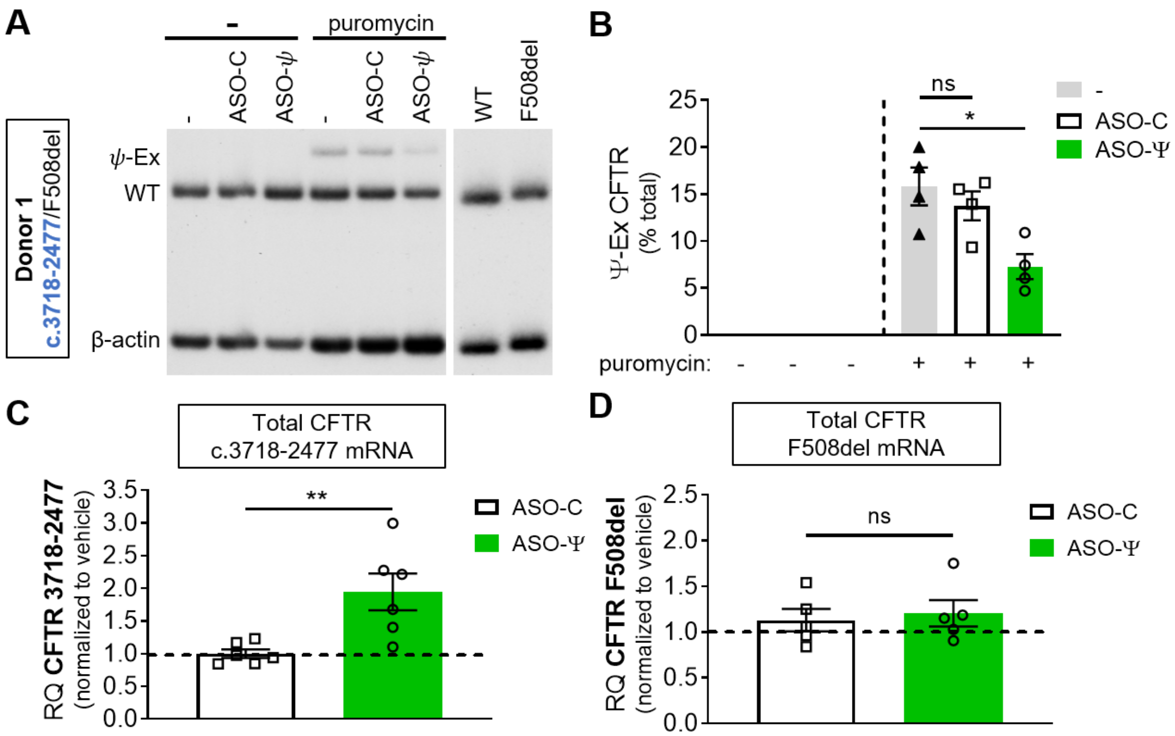

Supplementary Figure S5. ASO-ψ treatment increases full-length *CFTR* mRNA in compound heterozygous patient cells**.** (**A**) RT-PCR analysis of compound heterozygous hBE cells (c.3718-2477 T/F508del) treated as in Fig 3A. (**B**) ψ-Ex splicing quantified as the percent of total *CFTR*. (±SEM; N=4; One-way ANOVA with Tukey's multiple comparisons test, *p<0.05) (**C**) RT-qPCR analyses of total *CFTR* mRNA from the *CFTR* c.3718-2477C>T allele. (±SEM; N=6; t-test, **p<0.01) (**D**) F508del-*CFTR* isolated from the compound heterozygous patient (donor 1) treated as above (±SEM; N=5; t-test, ns=p>0.05). Relative quantity (RQ) was normalized to vehicle treatment.

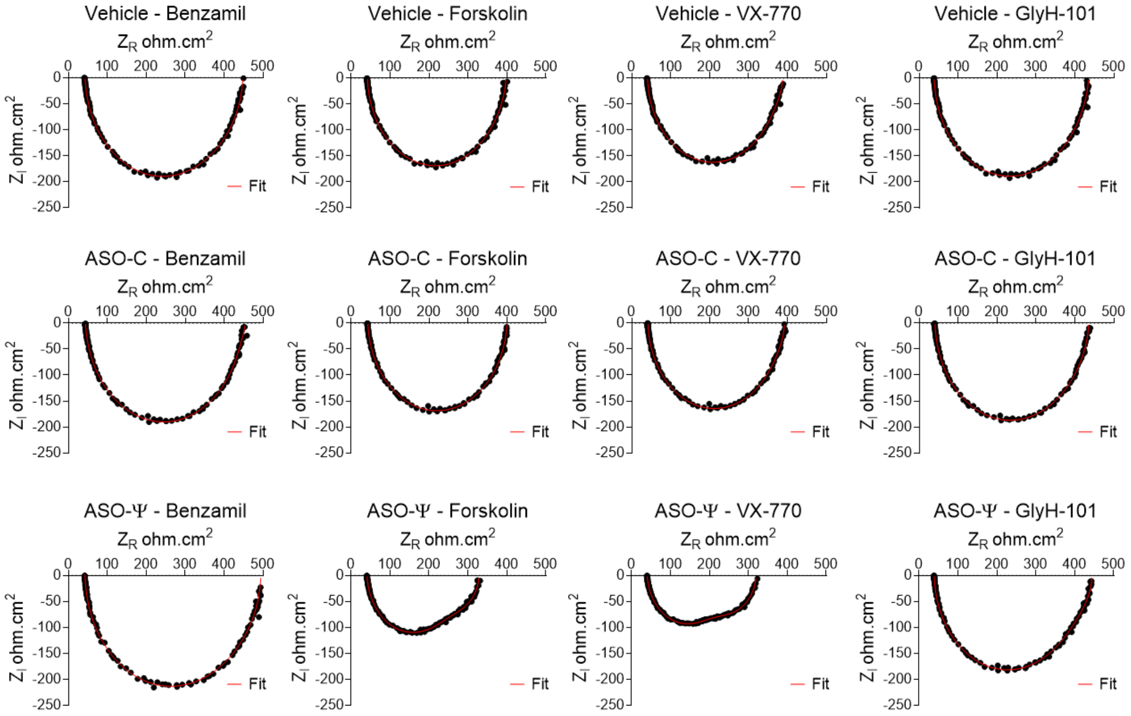

Supplementary Figure S6. Impedance spectra data presented as representative Nyquist plots from a single replicate after benzamil, forskolin, VX-770, and GlyH-101 treatments, in vehicle, ASO-C, or ASO-ψ treated homozygous *CFTR* c.3718-2477C>T hBE cells. Both the real (Z_R) and imaginary (Z_i) components of the measured impedance are plotted together from all 126 frequencies (black dots). Model fitting for each impedance spectrum is plotted as a red line (Fit).

ASOs	Sequence (5'-3')
ASO-Ψ	CCTTTCAGGGTGTCTTACTCACCAT
ASO-C	CCTCTTACCTCAGTTACAATTTATA
**Primers**	**Sequence (5'-3')**
hCFTRex22F	CCAAACCATACAAGAAT
hCFTRex24R	GATCACTCCACTGTTCAT
hGAPDHFor	GAAGGTGAAGGTCGGAGTC
hGAPDHRev	GAAGATGGTGATGGGATTTC
hβ-actinFor	AAAGACCTGTACGCCAACAC
hβ-actinRev	GTCATACTCCTGCTTGCTGAT
hSC351.7kbF	GGCGTGTATTGGAGCAGATGTA
hSC351.7kbR	CTGCTACACAACTGCGCCTTTT
hCFTR-3849F	CTTGTCATCTTGATTTCTGGAGAC
hCFTR-3849R	GGATCAGGGAAGAGTACTTTGT
hCFTR-F508F	TGGCACCATTAAAGAAAATATCATCTT
hCFTR-F508R	CTCAGTGTGATTCCACCTTCTC
hCFTR-DF508F	GGCACCATTAAAGAAAATATCATTGG
hCFTR-DF508R	CTCAGTGTGATTCCACCTTCT
**Probes**	**Sequence (5'-3')**
hCFTR-3849	/56-FAM/AAATGGTGG/ZEN/GCCTCTTGGGAAGAA/3IABkFQ/
hCFTR-F508	/56-FAM/ACAGAAGCG/ZEN/TCATCAAAGCATGCC/3IABkFQ/
hCFTR-DF508	/56-FAM/ACAGAAGCG/ZEN/TCATCAAAGCATGCC/3IABkFQ/

Supplementary Table S1 – Splice-switching Antisense Oligonucleotides, Primers, and Probes.

	Treatment:	Donor 1 N=9 **c.3718-2477/F508del**	Donor 2 N=3 **c.3718-2477/F508del**	Donor 3 N=4 **c.3718-2477**	Donor 4 N=6 **F508del**	Donor 5&6 N=6 **WT**
Baseline Rt (ohms.cm^2)	(-)	236.9 ± 19.28	205.6 ± 3.47	271.4 ± 9.09	300.9 ± 37.24	197.79 ± 20.17
	ASO-C	235.1 ± 19.08	205.2 ± 7.60	272.8 ± 8.02	299.8 ± 38.57	197.54 ± 11.99
	ASO-Ψ	215.9 ± 16.17	167.9 ± 11.60	240.0 ± 7.64*	287.1 ± 28.91	224.21 ± 15.76
	(-) C18	241.8 ± 18.19	226.2 ± 4.86	280.1 ± 9.86	303.0 ± 41.83	231.25 ± 39.66
	ASO-C C18	238.9 ± 19.98	214.8 ± 7.73	278.5 ± 5.10	303.2 ± 38.87	204.79 ± 23.90
	ASO-Ψ C18	219.6 ±17.51	172.8 ± 8.27*	247.2 ± 2.43*	292.9 ± 28.69	231.50 ± 23.26
Baseline Ieq (μA/cm^2)	(-)	141.2 ± 13.75	154.6 ± 3.46	109.2 ± 7.47	172.1 ± 16.73	58.74 ± 6.44
	ASO-C	149.6 ± 15.51	156.0 ± 2.64	110.8 ± 6.69	168.9 ± 16.90	58.89 ± 5.63
	ASO-Ψ	143.3 ± 12.09	160.0 ± 6.64	114.3 ± 7.20	161.7 ± 15.53	63.87 ± 4.65
	(-) C18	143.9 ± 14.00	153.9 ± 3.38	112.3 ± 8.69	177.0 ± 14.91	57.77 ± 6.78
	ASO-C C18	149.7 ± 16.26	167.7 ± 3.37	111.3 ± 5.98	173.5 ± 14.33	61.31 ± 3.91
	ASO-Ψ C18	152.3 ± 15.78	170.6 ± 2.62	110.0 ± 6.34	163.0 ± 14.21	59.47 ± 5.68
Benzamil Ieq (μA/cm^2)	(-)	8.8 ± 0.44	7.1 ± 0.13	7.3 ± 0.17	4.7 ± 0.83	15.34 ± 0.85
	ASO-C	9.3 ± 0.55	6.1 ± 0.15	7.4 ± 0.60	4.9 ± 0.37	14.97 ± 0.76
	ASO-Ψ	10.0 ± 0.46	8.1 ± 0.19	8.4 ± 0.49	5.0 ± 0.32	14.87 ± 1.28
	(-) C18	8.8 ± 0.62	7.3 ± 0.31	7.5 ± 0.46	5.1 ± 0.33	16.73 ± 0.57
	ASO-C C18	9.5 ± 0.54	8.0 ± 0.22	6.9 ± 0.38	5.1 ± 0.33	16.64 ± 1.03
	ASO-Ψ C18	10.3 ± 0.67	10.8 ± 0.94$^{+}_{*}$	8.8 ± 0.71	6.0 ± 0.39	18.30 ± 0.86
Forskolin Ieq (μA/cm^2)	(-)	17.5 ± 1.08	18.6 ± 0.39	18.5 ± 0.41	9.1 ± 0.83	34.72 ± 0.82
	ASO-C	21.2 ± 2.87	18.3 ± 0.46	18.5 ± 1.12	9.5 ± 0.91	34.92 ± 1.40
	ASO-Ψ	32.2 ± 2.70*	39.7 ± 0.21****	40.6 ± 0.67****	10.6 ± 0.38	38.03 ± 1.07
	(-) C18	22.6 ± 1.77	27.6 ± 0.61	20.9 ± 0.79	22.2 ± 0.85	34.13 ± 1.14
	ASO-C C18	25.2 ± 2.58	29.1 ± 0.93^{++++}	20.3 ± 0.71	22.8 ± 1.30^{++++}	34.98 ± 1.47
	ASO-Ψ C18	32.7 ± 2.49	48.4 ± 0.31$^{****}_{++++}$	44.5 ± 0.65****	27.2 ± 1.88^{++++}	37.79 ± 1.56
VX-770 Ieq (μA/cm^2)	(-)	20.5 ± 1.14	22.6 ± 0.46	20.7 ± 0.16	8.8 ± 1.26	34.57 ± 0.83
	ASO-C	24.1 ± 2.86	21.4 ± 0.45	20.5 ± 0.94	9.0 ± 1.25	34.58 ± 1.33
	ASO-Ψ	35.1 ± 2.55*	42.2 ± 0.77****	41.8 ± 1.14****	10.2 ± 1.11	37.81 ± 0.99
	(-) C18	26.6 ± 1.68	33.0 ± 0.55	21.6 ± 0.52	22.2 ± 0.43	33.94 ± 1.12
	ASO-C C18	28.7 ± 2.36	32.3 ± 0.67^{++++}	20.9 ± 0.44	22.4 ± 0.58^{++++}	34.76 ± 1.24
	ASO-Ψ C18	35.5 ± 2.56	50.5 ± 0.86$^{****}_{+++}$	43.7 ± 0.89****	27.1 ± 0.90$^{++++}_{*}$	37.49 ± 1.37
Bumetanide Ieq (μA/cm^2)	(-)	13.2 ± 0.64	11.5 ± 0.12	8.8 ± 0.09	3.9 ± 0.52	9.33 ± 1.49
	ASO-C	13.8 ± 0.72	11.3 ± 0.20	8.8 ± 0.67	4.2 ± 0.52	8.58 ± 1.27
	ASO-Ψ	15.5 ± 0.71	12.4 ± 0.39	9.9 ± 0.42	4.1 ± 0.29	9.64 ± 1.15
	(-) C18	13.1 ± 0.76	11.6 ± 0.19	8.2 ± 0.42	3.9 ± 0.53	8.06 ± 0.94
	ASO-C C18	13.8 ± 0.65	11.4 ± 0.22	7.7 ± 0.31	3.6 ± 0.39	8.65 ± 1.22
	ASO-Ψ C18	15.0 ± 0.69	13.3 ± 0.32	9.2 ± 0.52	4.5 ± 0.29	9.18 ± 0.79

*=ASO-C vs ASO-Ψ   *p<.05, **p<.01, ***p<.001, ****p<.0001

+=ASO alone vs ASO + C18 $^{+}$p<.05, $^{++}$p<.01, $^{+++}$p<.001, $^{++++}$p<.0001

Supplementary Table S2. I_{eq} and Rt values from donor HBE cell in Figures 2 and 6 and Supplementary Figures S2 and S3.

	Treatment:	Donor 3 N=3 **4 days** treated	Donor 3 N=3 **8 days** treated	Donor 3 N=3 **16 days** treated
Baseline Rt (ohms.cm^2)	(-)	205.5 ± 21.39	206.7 ± 21.18	285.8 ± 9.42
	ASO-C	183.0 ± 28.29	181.0 ± 21.76	299.7 ± 7.34
	ASO-Ψ	182.2 ± 26.52	183.7 ± 25.42	253.2 ± 11.46
Baseline Ieq (μA/cm^2)	(-)	119.5 ± 3.00	103.4 ± 6.79	100.3 ± 4.15
	ASO-C	114.9 ± 1.62	103.1 ± 5.90	95.0 ± 5.12
	ASO-Ψ	120.4 ± 4.09	95.3 ± 5.88	99.4 ± 4.44
Benzamil Ieq (μA/cm^2)	(-)	6.5 ± 1.04	5.4 ± 0.52	5.0 ± 0.80
	ASO-C	7.3 ± 1.15	5.7 ± 1.01	5.6 ± 0.35
	ASO-Ψ	10.9 ± 2.05	22.2 ± 8.96	16.48 ± 3.71*
Forskolin Ieq (μA/cm^2)	(-)	23.7 ±1.98	18.5 ± 1.45	14.7 ± 1.29
	ASO-C	26.0 ± 1.56	19.2 ± 1.05	15.3 ± 0.47
	ASO-Ψ	38.8 ± 2.62*	43.2 ± 2.29***	37.1 ± 2.56***
VX-770 Ieq (μA/cm^2)	(-)	24.5 ± 1.90	18.9 ± 1.31	14.4 ± 0.87
	ASO-C	27.3 ± 1.23	19.8 ± 1.00	15.2 ± 0.18
	ASO-Ψ	39.3 ± 2.41*	39.0 ± 3.38**	37.3 ± 2.68***
Bumetanide Ieq (μA/cm^2)	(-)	7.9 ± 1.47	5.8 ± 0.27	5.1 ± 0.74
	ASO-C	9.5 ± 1.29	5.8 ± 0.72	5.2 ± 0.14
	ASO-Ψ	8.6 ± 1.58	6.2 ± 1.80	6.4 ± 0.55

*=ASO-C vs ASO-Ψ   *p<.05, **p<.01, ***p<.001, ****p<.0001

Supplementary Table S3. I_{eq} and Rt values from donor 3 in Figure 4.
